# Prevention of medication-related osteonecrosis of the jaws secondary to tooth extractions. A systematic review

**DOI:** 10.4317/medoral.20963

**Published:** 2016-01-31

**Authors:** Márcio Diniz-Freitas, Jacobo Limeres

**Affiliations:** 1 Special Patients Unit. Medical-Surgical Dental Research Group (OMEQUI). Faculty of Medicine and Dentistry. University of Santiago de Compostela (Spain)

## Abstract

**Background:**

A study was made to identify the most effective protocol for reducing the risk of osteonecrosis of the jaws (ONJ) following tooth extraction in patients subjected to treatment with antiresorptive or antiangiogenic drugs.

**Material and Methods:**

A MEDLINE and SCOPUS search (January 2003 - March 2015) was made with the purpose of conducting a systematic literature review based on the Preferred Reporting Items for Systematic reviews and Meta-Analyses (PRISMA) guidelines. All articles contributing information on tooth extractions in patients treated with oral or intravenous antiresorptive or antiangiogenic drugs were included.

**Results:**

Only 13 of the 380 selected articles were finally included in the review: 11 and 5 of them offered data on patients treated with intravenous and oral bisphosphonates, respectively. No randomized controlled trials were found – all publications corresponding to case series or cohort studies. The prevalence of ONJ in the patients treated with intravenous and oral bisphosphonates was 6,9% (range 0-34.7%) and 0.47% (range 0-2.5%), respectively. The main preventive measures comprised local and systemic infection control.

**Conclusions:**

No conclusive scientific evidence is available to date on the efficacy of ONJ prevention protocols in patients treated with antiresorptive or antiangiogenic drugs subjected to tooth extraction.

**Key words:**Bisphosphonates, angiogenesis inhibitors, antiresorptive drugs, extraction, osteonecrosis.

## Introduction

Medication-related osteonecrosis of the jaws (ONJ) is defined as an area of exposed bone or bone that can be probed through an intra- or extraoral fistula in the maxillofacial region, persisting for over 8 weeks in patients receiving or who have received antiresorptive or antiangiogenic medication, and who have not undergone radiotherapy or present evidence of metastatic disease in the mentioned anatomical region (1). Once such lesions become established, their management is complicated and the course of the disease is difficult to predict – particularly in the most advanced cases (2). Prevention and control of the risk factors is therefore especially important. Osteonecrosis of the jaws may develop spontaneously or can be induced by invasive dental procedures (3), fundamentally tooth extractions (4,5). The exact prevalence of ONJ after tooth extraction is not clear, though according to the American Association of Oral and Maxillofacial Surgeons, two out of every three cases are related to oral surgery – particularly tooth extraction (6).

A number of perioperative measures have been proposed for preventing this complication, including antiseptic rinses immediately before extraction and until healing of the socket (7), antibiotic prophylaxis (8), alveoloplasty with primary closure (9), fibrin or autologous platelet-rich plasma (9), atraumatic extraction with orthodontic traction (10,11), ozone therapy (12), limitation of the number of extractions performed in each session (13), etc. Many of these proposed measures are fundamented upon personal experience and on consensus documents developed by scientific societies (1,6-9,13-20) ( Table 1), and their true efficacy is not known (21).

**Table 1:**
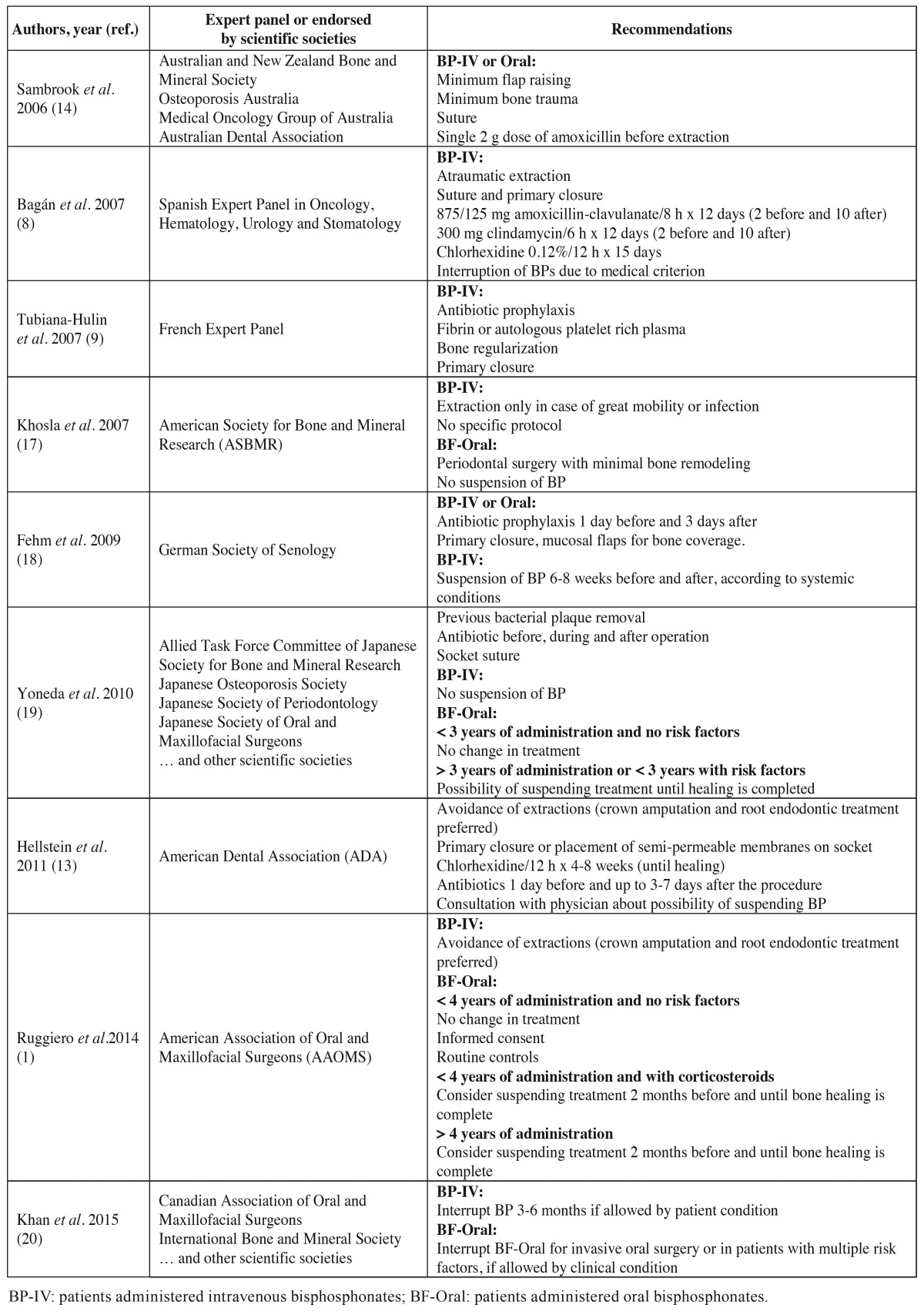
Principal protocols for the prevention of osteonecrosis of the jaws (ONJ) associated to the administration of antiresorptive or antiangiogenic drugs in patients subjected to oral surgery.

In view of the increasing number of patients receiving antiresorptive or antiangiogenic treatment, and the important morbidity associated to ONJ, we decided to conduct a systematic review with the purpose of identifying the most relevant protocols and best measures for preventing the development of ONJ secondary to tooth extraction.

## Material and Methods

The present systematic review was carried out following the Preferred Reporting Items for Systematic reviews and Meta-Analyses (PRISMA) guidelines (22). The PICO (Patient, Intervention, Comparison, Outcome) question that guided the review was: What is the most effective procedure for reducing the risk of ONJ after tooth extraction in patients receiving treatment with antiresorptive or antiangiogenic drugs?

- Search strategy

A systematic MEDLINE and SCOPUS database search (January 2003 - March 2015) was made to identify publications eligible for inclusion in the study, using a combination of MeSH terms and free text ( Table 2) as search strategy: “diphosphonates”, “bisphosphonates”, “antiresorptive”, “angiogenesis inhibitors” “angiogenesis”, “inhibitors”, “antiangiogenic”, “denosumab”, “sunitinib”, “tooth extraction”, “tooth”, “dental extraction”, “dental”, “extraction, “osteonecrosis”. As a complement to this search, we conducted a manual evaluation of articles included in the references of the identified full-text publications, with the selection of those citations considered to be of relevance.

**Table 2:**
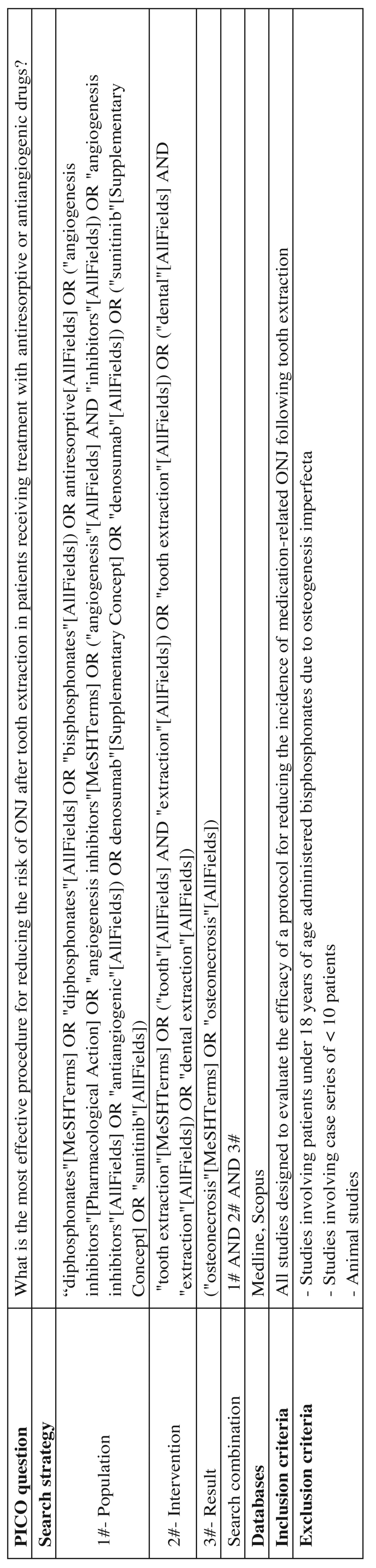
Systematic literature search strategy on the prevention of osteonecrosis of the jaws associated to the administration of antiresorptive or antiangiogenic drugs.

- Selection of studies

In order to assess the eligibility of the studies, two reviewers (MD, JL) traced the titles and abstracts of the publications generated by the search strategy. The full-text articles were retrieved in the case of those studies that appeared to satisfy the screening criteria, and in the case of those which offered too little information in the title / abstract to firmly decide inclusion of the study or not. All articles contributing information on tooth extractions in patients treated with oral or intravenous antiresorptive or antiangiogenic drugs were included.

The selected studies assessed the efficacy of a protocol for reducing the incidence of ONJ after tooth extraction, and were required to supply information on the type of antiresorptive or antiangiogenic treatment used, the administration route (intravenous or oral), the indication of treatment, a clear definition of the presence of ONJ, and the duration of follow-up (in months). The articles also were required to clearly specify the prevention protocol employed (surgical technique, type and dose of antibiotic administered, etc.).

Studies in patients under 18 years of age administered bisphosphonates (BPs) due to osteogenesis imperfecta were excluded, as were case series involving fewer than 10 patients, and animal studies. In the case of studies involving expansions of the same series of patients, only the most recent data were considered.

The two reviewers independently assessed compliance with the inclusion and exclusion criteria. The reason for exclusion was recorded in the case of those articles that were eliminated in this phase.

- Data extraction

The identified references were processed using the bibliographic management program Refworks (Proquest), and the data extracted from the articles were entered in a MS Excel spreadsheet. The studies were divided into three groups: (a) studies involving patients treated with intravenous BPs; (b) studies involving patients treated with oral BPs; and (c) studies involving patients treated with other antiresorptive or antiangiogenic drugs. The main variables analyzed in each study were: authors, date of publication, sample size, drug type, dose and administration route, indication and, number of extractions and their location (maxilla or mandible), the preventive protocol employed (antibiotic prophylaxis, surgical technique and local measures), and the appearance of ONJ.

## Results and Discussion

**Figure 1:**
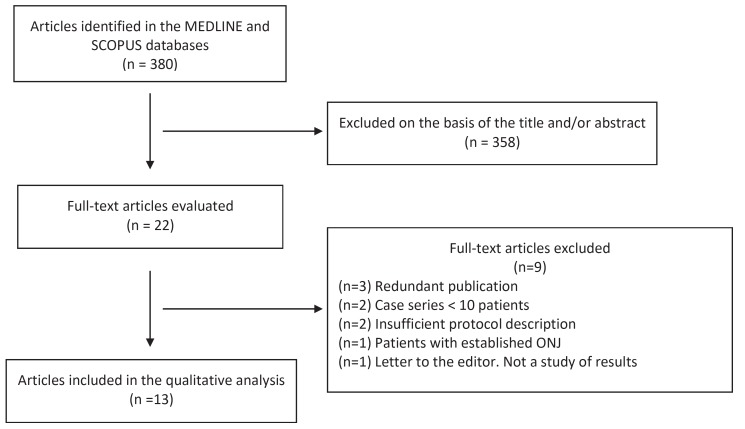
Schematic representation of the publication screening and inclusion process.

The article screening process is schematically shown in figure 1. Following the systematic search and the elimination of duplicate publications, we identified a total of 380 articles, of which 358 were discarded after assessing the title or abstract. Of the remaining 22 full-text articles, we eliminated 9 that failed to meet the inclusion criteria (Fig. 1), leaving a final total of 13 publications. Of these, 11 and 5 offered data on patients treated with intravenous and oral BPs, respectively ( Table 3,  Table 3 continue ,  Table 4). No articles on the prevention of ONJ in patients treated with other antiresorptive or antiangiogenic drugs were included. All the included publications were case series or cohort studies; we found no randomized controlled trials.

**Table 3:**
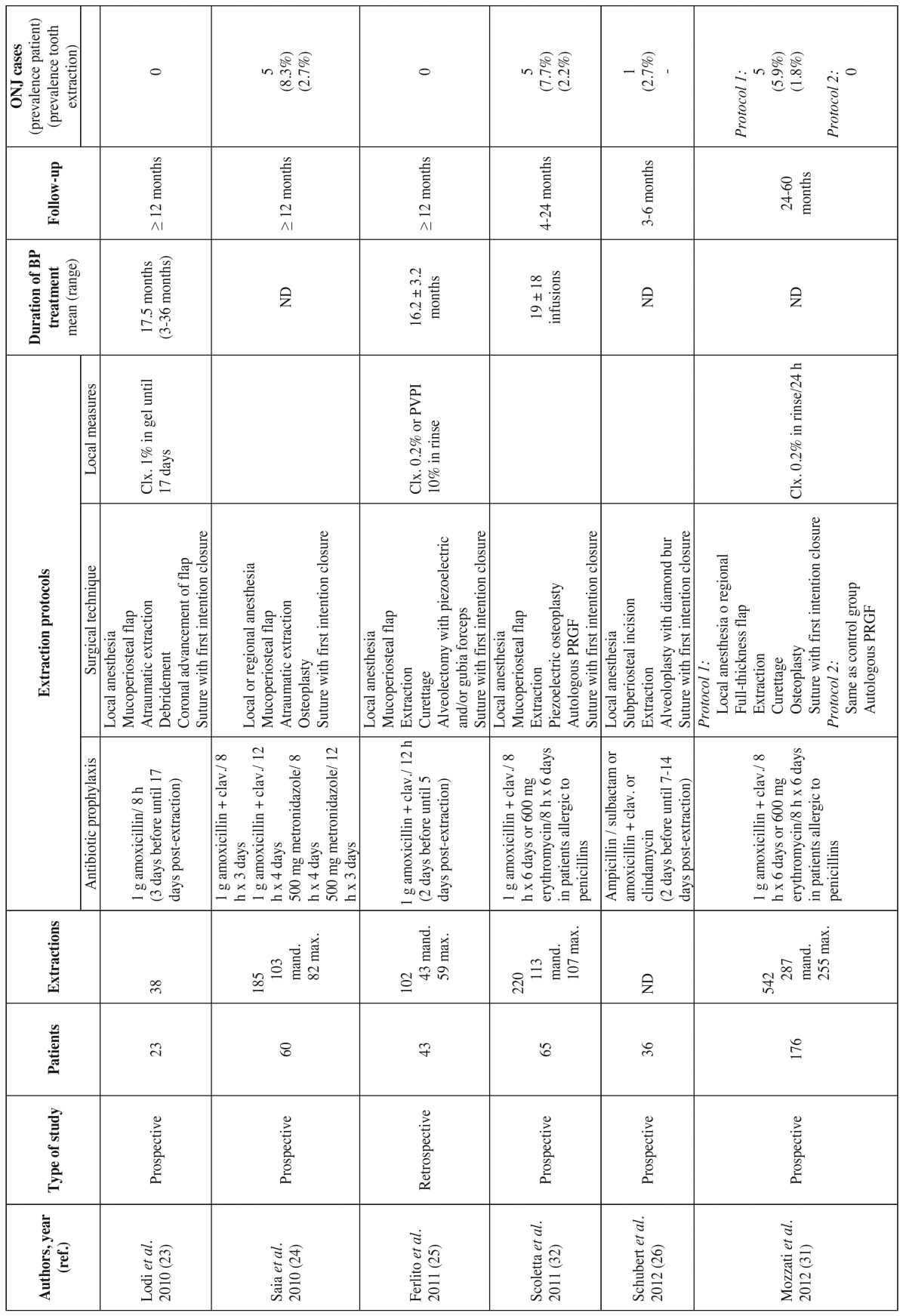
The most relevant studies on the prevention of osteonecrosis of the jaws (ONJ) associated to intravenous bisphosphonate treatment in patients subjected to tooth extraction.

**Table 3 continue:**
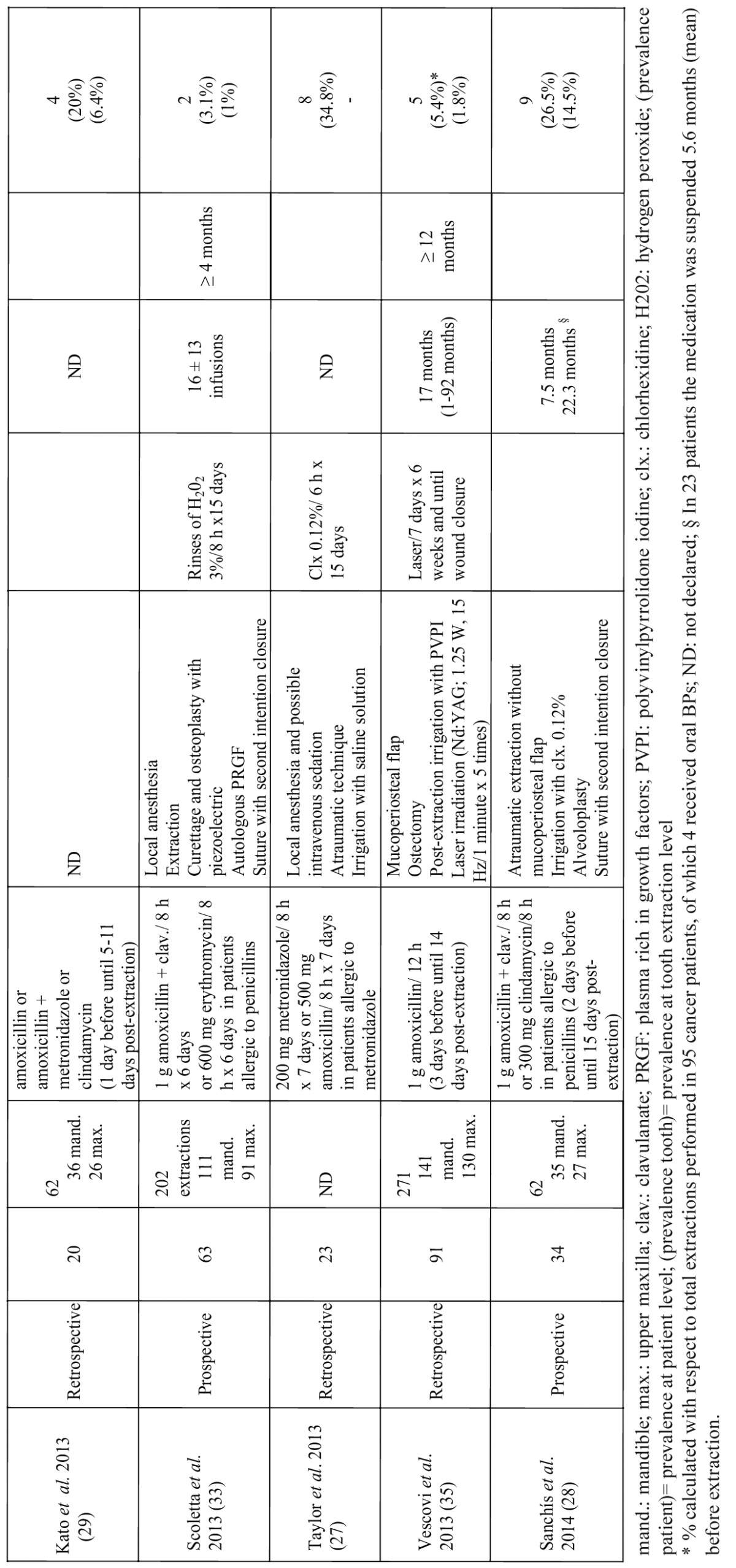
The most relevant studies on the prevention of osteonecrosis of the jaws (ONJ) associated to intravenous bisphosphonate treatment in patients subjected to tooth extraction.

**Table 4:**
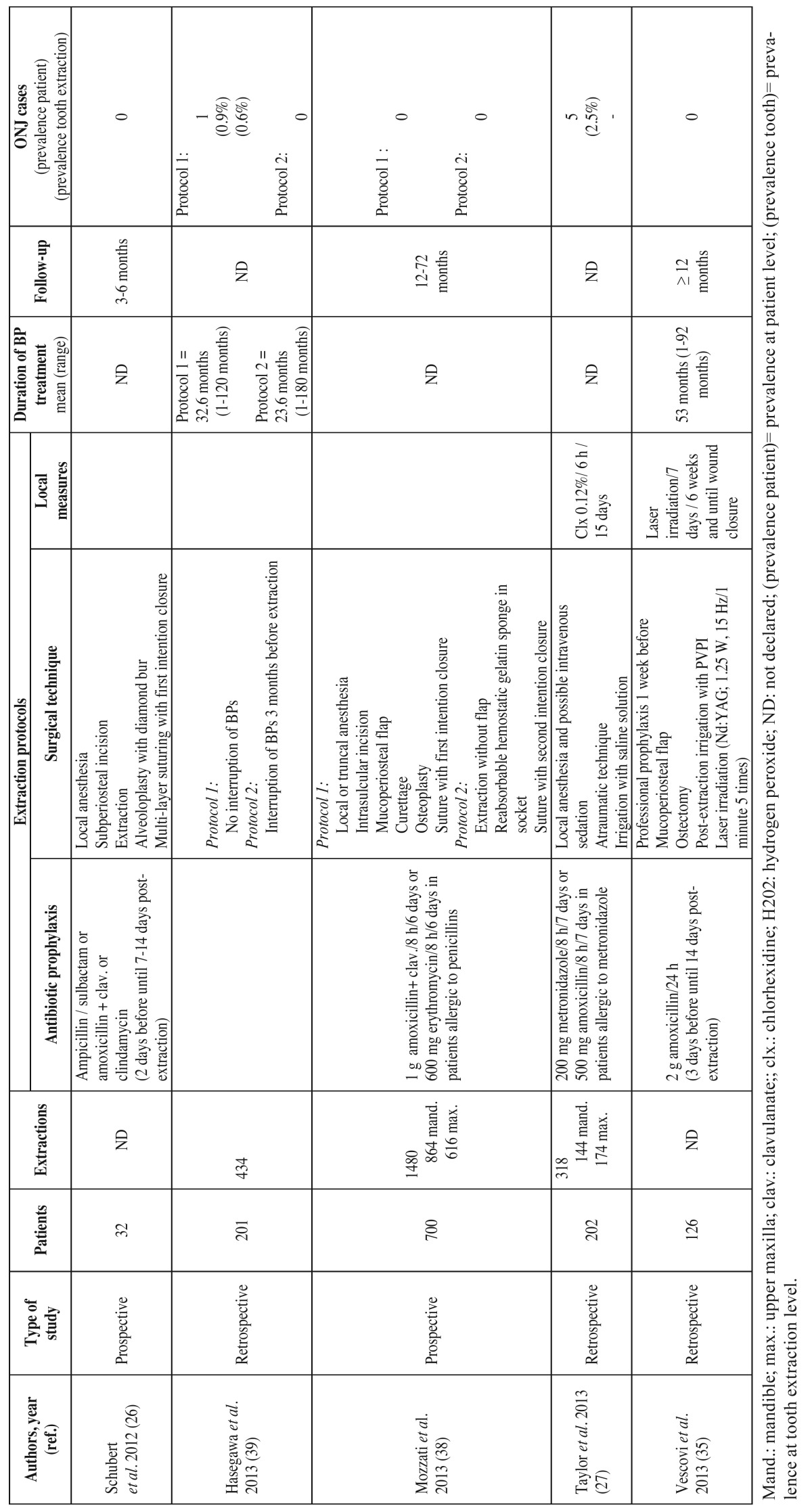
The most relevant studies on the prevention of osteonecrosis of the jaws (ONJ) associated to oral bisphosphonate treatment in patients subjected to tooth extraction.

The studies involving subjects treated with intravenous BPs included a total of 634 patients, with a prevalence of ONJ of 6,9% (range 0-34.7%). In turn, the studies involving oral BPs included a total of 1261 patients, with a prevalence of ONJ of 0.47% (range 0-2.5%). The main preventive measures comprised local and systemic infection control ( Table 3,  Table 3continue , Table 4).

Although medication-related ONJ may manifest spontaneously, in up to 80% of all cases it is associated to tooth extractions or other surgical procedures involving bone exposure. In this study we therefore considered extraction as a potential risk factor for ONJ. Our findings show that few authors have systematically applied any of the prophylactic protocols proposed by the different international expert committees or scientific societies (1,6-9,13-20). Most articles on the efficacy of preventive measures before tooth extraction in patients treated with antiresorptive or antiangiogenic drugs have methodological shortcomings, are not randomized or controlled, involve an insufficient sample size, and apply very heterogeneous preventive protocols – combining common sense initiatives such as antibiotic treatment with other much more sophisticated strategies such as platelet rich plasma or low-power laser irradiation. This heterogeneity and the limitations of the reviewed studies therefore do not allow quantitative analysis (meta-analysis).

- Bisphosphonates via the intravenous route

Lodi *et al*. (23) were probably the first authors to propose a specific protocol for tooth extraction in patients treated with intravenous BPs, based on local and systemic infection control measures. These investigators conducted a prospective study of 23 patients subjected to 38 extractions, and identified no cases of ONJ over a minimum follow-up period of 12 months. In view of these results, they concluded that the conduction of a randomized, placebo-controlled trial without the application of local and systemic infection control measures would not be ethically acceptable.

Following the work of Lodi *et al*. (23), new both retrospective and prospective studies were made, applying local and systemic infection control measures. In these publications the prevalence of ONJ varied between 0-23.5% (24-29). Shared features of these studies were the provision of antibiotic prophylaxis; atraumatic tooth extraction with the raising of a flap to allow first-intention closure and healing, minimizing direct contact of the oral bacteria with the socket; and the local application of antiseptic products. However, the composition of the antimicrobials used, the dosage and the duration of treatment varied considerably ( Table 3, Table 3continue). Although randomized trials would be needed to determine the true efficacy of antibiotic prophylaxis in patients subjected to extraction and treated with antiresorptive or antiangiogenic drugs, antibiotics do appear to exert a certain preventive effect, as demonstrated by some studies in animals (30) and retrospective studies in patients with multiple myeloma (5). Mozzati *et al*. (31) conducted a prospective study of 176 patients subjected to a total of 542 extractions, and randomized to two groups: in one group surgery involved the raising of a mucoperiosteal flap to allow first-intention closure and healing, while in the other group extraction was carried out based on the same protocol but placing plasma rich in growth factors in the socket before first-intention closure. After a follow-up period of between 24-60 months, they recorded 5 cases of ONJ (1.8%) in the first group and none in the second.

Scoletta *et al*. (32) proposed the use of autologous plasma rich in growth factors associated to systemic antibiotic treatment in order to accelerate the healing process. In their initial protocol, the authors raised a mucoperiosteal flap to allow healing by first intention. In a prospective study involved 65 patients subjected to 220 extractions, they documented ONJ in 7.6% of the cases (representing 2.2% of the extractions made). These same authors subsequently improved their protocol, eliminating the mucoperiosteal flap and performing cross-suturing over the socket in order to maintain the stability of the plasma rich in growth factors (33). With this new protocol, the authors were able to reduce the prevalence of ONJ to 1.5% of the patients and 0.9% of the extractions - in addition to simplifying the technique and shortening the surgery time. In a recent meta-analysis on the role of autologous platelet concentrates in the prevention and treatment of ONJ related to BPs, Del Fabbro *et al*. (34) concluded that although the published evidence is still weak, these products might offer benefits in terms of the prevention of ONJ in patients undergoing oral surgery.

Recently, Vescovi *et al*. (35) proposed a protocol involving low-power laser irradiation associated to antibiotic prophylaxis. Specifically, the proposal included Nd:YAG laser biostimulation immediately after extraction and then on a weekly basis until 6 weeks or closure of the surgical wound. On applying this protocol to a series of 91 cancer patients receiving intravenous BPs, the authors recorded ONJ in 5 patients (5,5%)(representing 1.8% of the 271 extractions made).

- Bisphosphonates via the oral route

The relationship between intravenous BPs and ONJ is fundamented upon solid epidemiological evidence, though the association between ONJ and oral BPs has been subject to strong controversy. Nevertheless, recent studies appear to offer tangible evidence of such an association (36). Since osteoporotic patients require prolonged treatment, the cases of ONJ related to oral BPs have increased, and a recent retrospective multicenter study suggests that the relative frequency of ONJ in osteoporotic patients treated with oral BPs is greater than previously believed (37). In the same way as in intravenous bisphosphonate therapy, the strategies proposed for preventing ONJ in patients receiving oral BPs are based on local and systemic infection control ( Table 4). Mozzati *et al*. (38) conducted a prospective study of 700 patients subjected to a total of 1480 extractions, randomized to two groups: in one group surgery involved the raising of a mucoperiosteal flap to allow first-intention closure and healing, while in the other group extraction was carried out without the raising of a flap and placing reabsorbable hemostatic sponge material in the socket to protect the wound. After 12-72 months of follow-up, no cases of ONJ were observed in either group.

The drug holiday concept (temporary suspension of the medication) in patients receiving oral BPs have been the subject of debate (1). The consensus document published by the American Association of Oral and Maxillofacial Surgeons in 2006 recommended interruption of the treatment from three months before to three months after extraction, if allowed by the systemic conditions of the patient (6). The 2014 update on this document (1) reduced the drug holiday period before extraction to two months, with application of this protocol only to patients who had received BPs for over four years. Hasegawa *et al*. (39) in turn conducted a retrospective study of 201 patients treated with oral BPs and subjected to a total of 434 tooth extractions. The patients were randomized to two groups: in one group oral BPs were suspended for three months before extraction, while no treatment interruption was applied in the other group. The authors identified a single case of ONJ in the latter group (0.6%) and none in the group in which oral BPs were temporarily suspended.

There is no evidence that the interruption of oral BPs is able to eliminate the risk of ONJ. On the other hand, temporary suspension of the medication may have a negative impact in terms of bone resorption. It is therefore necessary to consider the risks of the dental procedure and discuss the possibility of suspending antiresorptive treatment with the prescribing physician (13). Furthermore, in our setting, a significant percentage of patients receiving oral BPs and who visit the dentist for extractions do not meet the criteria for prescribing bisphosphonate therapy (40). As a result, patient re-evaluation by the physician should be considered, along with possible suspension of the treatment before dental surgery is carried out.

Regarding other prophylactic measures, Vescovi *et al*. (35), on using the laser treatment described above in relation to patients receiving intravenous BPs, recorded no cases of ONJ in 126 patients administered oral BPs and subjected to tooth extraction.

No conclusive scientific evidence is available to date on the efficacy of ONJ prevention protocols in patients subjected to tooth extraction and treated with antiresorptive or antiangiogenic drugs. In practical terms, and until future studies are able to define the ideal protocol, adoption of the preventive measures proposed by the international expert committees has weak scientific justification, but could afford some coverage from the medical-legal perspective.
